# Prediction of significant congenital heart disease in infants and children using continuous wavelet transform and deep convolutional neural network with 12-lead electrocardiogram

**DOI:** 10.1186/s12887-025-05628-2

**Published:** 2025-04-24

**Authors:** Yu-Shin Lee, Hung-Tao Chung, Jainn-Jim Lin, Mao-Sheng Hwang, Hao-Chuan Liu, Hsin-Mao Hsu, Ya-Ting Chang, Syu-Jyun Peng

**Affiliations:** 1 Division of Cardiology, Department of Pediatrics, Chang Gung Memoral Hospital Linkou Branch, Taoyuan, Taiwan; 2 In-Service Master Program in Artificial Intelligence in Medicine, College of Medicine, Taipei Medical University, No.250, Wuxing St., Xinyi Dist., Taipei City, 110 Taiwan; 3 Division of Pediatric Intensive Care, Department of Pediatrics, Chang Gung Memorial Hospital, Linkou Branch, Taoyuan, Taiwan; 4 Clinical Big Data Research Center, Taipei Medical University Hospital, Taipei Medical University, Taipei, Taiwan

**Keywords:** Congenital heart disease, Electrocardiogram, Artificial intelligence, Continuous wavelet transformation, Cardiac screening

## Abstract

**Background:**

Congenital heart disease (CHD) affects approximately 1% of newborns and is a leading cause of mortality in early childhood. Despite the importance of early detection, current screening methods, such as pulse oximetry and auscultation, have notable limitations, particularly in identifying non-cyanotic CHD. (AI)-assisted electrocardiography (ECG) analysis offers a cost-effective alternative to conventional CHD detection. However, most existing models have been trained on older children, limiting their generalizability to infants and young children. This study developed an AI model trained on real-world ECG data for the detection of hemodynamically significant CHD in children under five years of age.

**Methods:**

ECG data was retrospectively collected from 1,035 patients under five years old at Chang Gung Memorial Hospital, Taoyuan, Taiwan (2013–2020). Based on ECG findings, patients were categorized into the following groups: normal heart structure (NOR), non-significant right heart disease (RHA), significant right heart disease (RHB), non-significant left heart disease (LHA), and significant left heart disease (LHB). ECG signals underwent preprocessing using continuous wavelet transformation and segmentation into 2-s intervals for data augmentation. Transfer learning was applied using three pre-trained deep learning models: ResNet- 18, InceptionResNet-V2, and NasNetMobile. Model performance was evaluated in terms of accuracy, sensitivity, specificity, F1 score, and area under the receiver operating characteristic curve (AUC).

**Results:**

Among the tested models, the model based on ResNet-18 demonstrated the best overall performance in predicting clinically significant CHD, achieving accuracy of 73.9%, an F1 score of 75.8%, and an AUC of 81.0% in differentiating significant from non-significant CHD. InceptionResNet-V2 performed well in detecting left heart disease but was computationally intensive. The proposed AI model significantly outperformed conventional ECG interpretation by pediatric cardiologists (accuracy 67.1%, sensitivity 71.6%).

**Conclusions:**

This study highlights the potential of AI-assisted ECG analysis for CHD screening in young children. The ResNet-18-based model outperformed conventional ECG evaluation, suggesting its feasibility as a supplementary tool for early CHD detection. Future studies should focus on multi-center validation, inclusion of more CHD subtypes, and integration with other screening modalities to improve diagnostic accuracy and clinical applicability.

**Supplementary Information:**

The online version contains supplementary material available at 10.1186/s12887-025-05628-2.

## Background

Roughly 1% of all newborns present with congenital heart disease (CHD) [1, 2], and most CHD-related mortalities occur before the age of five, making early detection crucial. Echocardiography is the gold standard for diagnosing CHD; however, it is an expensive procedure requiring highly trained personnel. Several methods have been devised for CHD detection. Pulse oximetry performed at 24 h after birth is a cost-effective alternative for the screening of critical cyanotic congenital heart conditions that require intervention [3]. One meta-analysis reported that this method achieves sensitivity of 76.3% with specificity of 99.9% and a false positive rate of 0.14% [4]. However, this method fails to detect common noncyanotic CHDs, such as ventricular septal defect (VSD) and patent ductus arteriosus (PDA) [3]. Left heart disease can have a profound impact on hemodynamics, leading to early heart failure. Companion screening methods are required to enhance sensitivity to noncyanotic CHDs.

Auscultation for the detection of heart murmurs is the method most commonly used by pediatricians in screening for major CHDs. Detecting heart murmurs of > grade 2 has been shown to yield sensitivity of 89.6% with specificity of 97.3% and a false positive rate of 2.7% [5]. While heart murmur analysis is effective in dealing with most CHDs associated with left heart disease, it is far less effective in detecting CHDs associated with right heart disease, such as atrial septal defects, which often do not produce heart murmurs [6]. Moreover, access to trained pediatricians is often limited to major medical centers, and the associated costs are very high. There is a demand for screening tools applicable to a broader range of CHDs.

Researchers have made significant strides in applying artificial intelligence (AI) to the interpretation of phonocardiograms (PCG) for the detection of common CHDs. Some models have achieved sensitivity of 99.0% with specificity of 98.0% and a false positive rate of 2.0% [7]. However, these preliminary studies were based on well-prepared datasets, raising concerns about the practical applicability of this method under real-world conditions.

Electrocardiogram (ECG) analysis is another method commonly used for the screening of congenital heart disease. This affordable method provides objective measurements of electrical activity, avoiding the subjective interpretation of indistinct indicators by clinicians. ECG also enables the detection of conditions that do not produce an audible heart sound, such as atrial septal defect (ASD), where ECG indicators occur earlier than heart murmurs.

Table 1 lists representative studies on the screening of congenital heart disease [4, 7–13]. One AI model demonstrated good performance in detecting hemodynamic atrial septal defect (Qp/Qs > 1.5) with sensitivity of 76%, specificity of 96% and a false positive rate rate of 2.0% in school-aged children [8]. Another AI model trained using a large ECG database for the detection of CHD demonstrated sensitivity of 74.7% and specificity of 94.1% [14].

**Table 1:** Representative literature on the screening of congenital heart disease

Study	Study Population	Methodology	Key Results	Limitations
Plana et al. (2018) [4]	Meta-analysis (*n* = 457,202)	Pulse oximetry	Sensitivity: 76.3% Specificity: 99.9%	Detected only critical cyanotic heart disease
Lv et al. (2021) [9]	1,362 CHD children requiring surgery	AI based heart sound analysis	Accuracy: 98% Sensitivity: 91% Specificity: 97%	Unable to identify CHDs without significant murmur
Xu et al. (2022) [10]	Children aged 2 days to 12 years (408 CHD, 553 controls)	AI-based heart sound analysis	Accuracy: 95% Sensitivity: 94% Specificity: 96%	Detected CHD with heart murmurs, while overlooking CHDs without murmur
Liu et al. (2022) [11]	Children (475 CHD, 409 controls)	AI-based heart sound analysis	Accuracy: 83% ASD detection accuracy: 65%	Poor performance in ASD detection, due to variations in ECG caused by factors, such as right heart pressure and defect size
Alkahtani et al. (2024) [7]	583 PCG from local database and 23 ECG from public database	AI-based heart sound analysis	Accuracy: 98.6% Sensitivity: 99.0% Specificity: 98.0%	Binary classifications (Normal vs. abnormal). Patient age not specified
Du et al. (2020) [12]	68,969 ECGs (58,624 Non-CHD and 10,345 CHD)	AI-based ECG analysis	Sensitivity: 74.7% Specificity: 94.1%	Demographic of CHD patients not specified. Patient age not specified
Mori et al. (2021) [8]	Children aged 6–18 (364 ASD, 828 normal)	Deep learning-based ECG analysis	Accuracy: 0.89 Specificity: 0.96 F1 Score: 0.81	Focus on school-aged children Included only ASD patients
Liu et al. (2023) [13]	Adults (1,196 ASD, 21,430 controls)	AI-based ECG analysis	Accuracy: 0.86 Specificity: 0.87 AUC: 0.88	Focus on adults with no data from infants or young children. Did not address hemodynamics

It is important to note that most of the training data assembled for these models was from school-aged children and adolescents [12]. Moreover, many of these studies did not include demographic data or address the issue of hemodynamic significance.

There is a pressing need for affordable and accessible screening methods for the detection of non-critical, but clinically significant CHDs in early childhood. This study trained an AI model using real-world ECG data to detect the presence of clinically significant CHDs in children under the age of five.

## Participants and methods

### Data sources

Patient data were collected retrospectively from Chang Gung Memorial Hospital, Taiwan. The study included patients under five years old who were diagnosed with specific CHDs, including atrial septal defect (ASD), ventricular septal defect (VSD), patent ductus arteriosus (PDA), pulmonary stenosis (PS), aortic stenosis (AS), coarctation of the aorta (CoA), and Tetralogy of Fallot (TOF) between January 2013 and December 2020. Data were also collected from patients under five years old who had normal heart structure (confirmed by ECG) and visited the outpatient department between December 2020 and March 2021.

### Patient grouping process

**Fig. 1:**
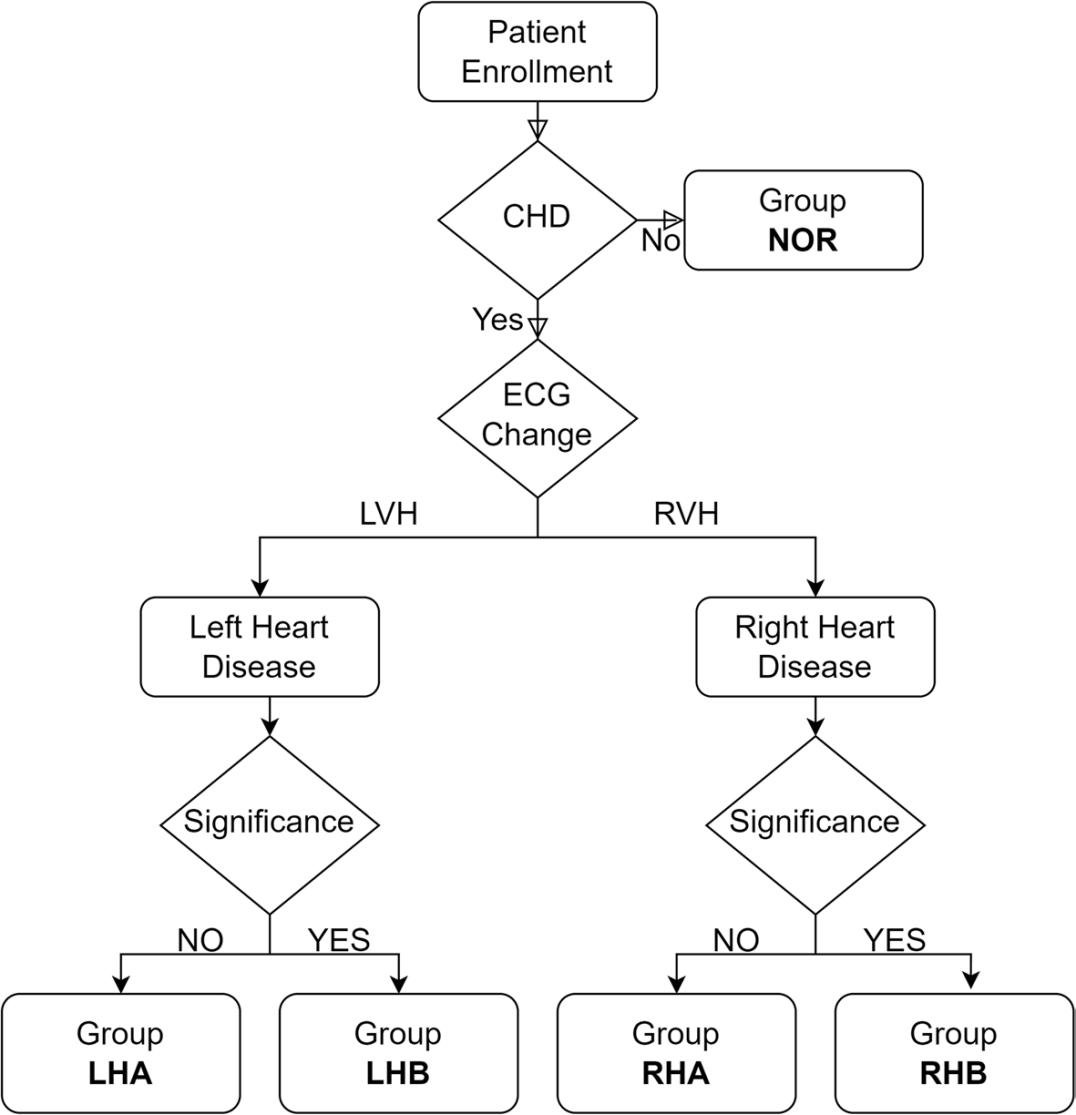
Flowchart illustrating the process of classifying infants and children into five groups based on the characteristics of congenital heart disease (CHD) and hemodynamic significance: normal heart structure (NOR), non-significant left heart disease (LHA), significant left heart disease (LHB), non-significant right heart disease (RHA), and significant right heart disease (RHB)

Initial patient enrollment (Fig. 1) was followed by classification based on the presence of congenital heart disease (CHD). Patients without CHD were categorized into the NOR (normal) group. Those diagnosed with CHD underwent further stratification based on electrocardiographic (ECG) signals. Patients with CHDs causing left ventricular hypertrophy (LVH) were classified under Left Heart Disease (LHD), while those with patients with CHDs that causing right ventricular hypertrophy (RVH) were categorized as Right Heart Disease (RHD). Within these groups, disease severity was further assessed according to clinical significance. The criteria for determining clinical significance are outlined in Table 2.

**Table 2:** Significance criteria for common congenital heart diseases

Screening Category	Condition	Significant Impact	Significance Criteria (Potential for Further Evaluation or Treatment)
Normal Cardiac Structure	——	——	——
Right Heart Diseases	Atrial Septal Defect (ASD)	No	Lesion < 5 mm
		Yes	Lesion ≥ 5 mm or Right heart enlargement
	Pulmonary Stenosis (PS)	No	Trans-pulmonary valve Vmax < 3.0 m/s
		Yes	Trans-pulmonary valve Vmax ≥ 3.0 m/s (Potential need for further evaluation)
	Tetralogy of Fallot (TOF)	Yes	Echocardiographic report confirms TOF diagnosis
Left Heart Diseases	Ventricular Septal Defect (VSD)	No	Lesion < 4 mm
		Yes	Lesion ≥ 4 mm or Left heart enlargement
	Patent Ductus Arteriosus (PDA)	No	Lesion < 2 mm
		Yes	Lesion ≥ 2 mm or Hemodynamically significant impact
	Aortic Stenosis (AS)	No	Trans-aortic valve Vmax < 3.0 m/s
		Yes	Trans-aortic valve Vmax ≥ 3.0 m/s (Potential need for further evaluation)
	Coarctation of the Aorta (CoA)	No	Trans-aortic flow velocity < 3.0 m/s
		Yes	Trans-aortic flow velocity ≥ 3.0 m/s (Potential need for further evaluation)

Below, we present the criteria used for classifying congenital heart diseases according to severity and assessing clinical significance:

- Atrial Septal Defect (ASD) [15]: Defects measuring < 5 mm were classified as small lesions with an 87% likelihood of spontaneous closure, typically managed by observation and regular follow-up.
- Pulmonary Valve Stenosis (PS) [16]: Echocardiographic findings indicating a pulmonary artery flow velocity of < 3 m/s were categorized as mild pulmonary stenosis, typically managed by observation and follow-up.
- Tetralogy of Fallot (TOF): A common cyanotic congenital heart disease with a significant impact on cardiac function and infant growth, necessitating corrective surgery.
- Ventricular Septal Defect (VSD) [17]: Small defects (< 4 mm) are more likely to undergo spontaneous closure than are larger defects.
- Patent Ductus Arteriosus (PDA) [18]: Small lesions (< 2 mm) can be treated via transcatheter coil occlusion. Larger lesions (> 2 mm) can have a significant impact on hemodynamics, requiring closure via transcatheter device placement.
- Aortic Valve Stenosis (AS) [19]: An aortic flow velocity of > 3 m/s indicates moderate aortic stenosis, which can impair ventricular diastolic function.
- Coarctation of the Aorta (CoA) [20]: A flow velocity of roughly 3 m/s corresponds to a pressure gradient of ~ 40 mmHg, which is associated with significant coarctation, as verified by cardiac catheterization and angiography.

Patients in the LHD group were divided into two subgroups: those with non-significant left heart disease (LHA) and those with significant left heart disease (LHB). Similarly, patients in the RHD group were classified into RHA (non-significant right heart disease) and RHB (significant right heart disease). The grouping methodology used in this study is illustrated in Fig. 1.

### Model construction

To develop a machine learning model capable of detecting CHDs of clinical significance, we established the following classification frameworks based on ECG data:

1. Right heart disease:
  ◦ Model 1: NOR vs. (RHA + RHB) – To predict the presence of total right heart disease (RHD).
  ◦ Model 2: (NOR + RHA) vs. RHB – To predict the presence of significant right heart disease (RHB).
2. Left heart disease:
  ◦ Model 3: NOR vs. (LHA + LHB) – To predict the presence of total left heart disease (LHD).
  ◦ Model 4: (NOR + LHA) vs. LHB – To predict the presence of significant left heart disease (LHB).
3. Significant CHD:
  ◦ Model 5: (NOR + RHA + LHA) vs. (RHB + LHB) To predict congenital heart disease of clinical significance.

### Data acquisition

For each patient, 12-lead resting ECG signals were recorded using a GE MAC 5500 HD device (GE Healthcare, Chicago, Illinois, USA). ECGs were collected in a calm and resting state to minimize motion artifacts. Asmall group of senior technicians (> 20 years of experience) performed all ECG acquisitions to ensure consistency. ECG signals were recorded at a sampling rate of 500 Hz for 10 s and stored in XML format within the MUSE Cardiology Information System.

To facilitate analysis, the XML files were converted to CSV format using Python within the Anaconda Prompt environment (Austin, Texas, USA). The CSV files were subsequently imported into MATLAB 2022b (Natick, Massachusetts, USA), where they underwent continuous wavelet transformation (CWT) based on the Morlet wavelet to generate time–frequency spectrograms. The transformed spectral data were then saved in MAT format for further processing.

To maximize data utilization, each 10-s ECG recording was segmented into five 2-s overlapping segments, thereby increasing the number of samples. Each 2-s segment was represented as a 12 × 1000 matrix, with each row corresponding to one of the 12 ECG leads (I, II, III, aVR, aVL, aVF, V1, V2, V3, V4, V5, and V6) and each column representing a time point.

Each ECG segment was labeled according to patient classification (Normal, Non-significant Right Heart Disease, Significant Right Heart Disease, Non-significant Left Heart Disease, Significant Left Heart Disease). All data were cross-checked by pediatric cardiologists to ensure data quality and correct labeling. Preprocessed ECG data and spectrograms were stored in MAT format for model training and analysis.

**Fig. 2:**
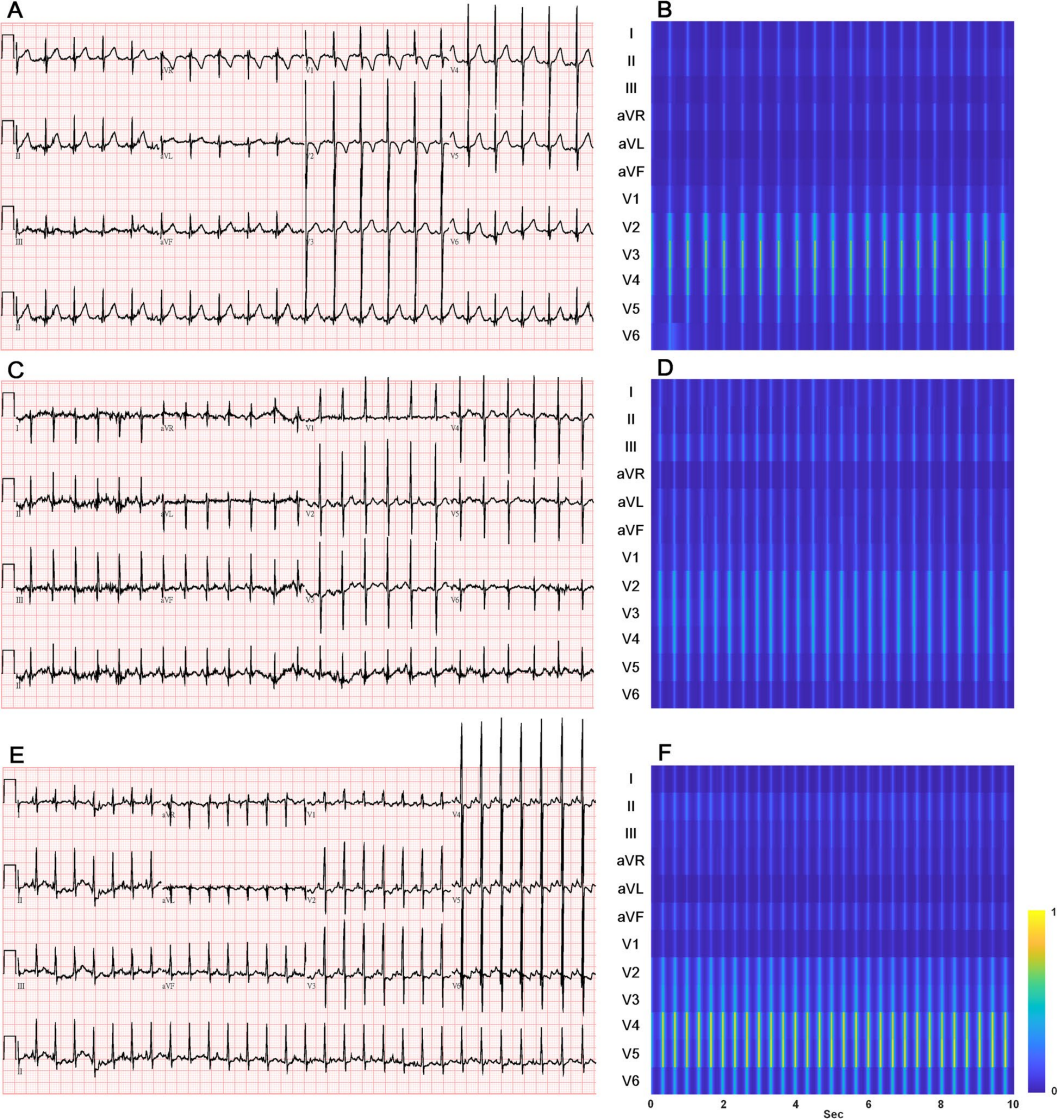
Examples of 12-lead ECG and corresponding continuous wavelet transform time-frequency diagrams (upper limit of 2 × 10^5^): (2 A) infant with normal heart structure with (2B) corresponding time-frequency diagram; (2 C) infant with right heart disease (tetralogy of Fallot) with (2D) corresponding time-frequency diagram, where leads V2 and V3 exhibit signals of the highst intensity; (2E) infant with left heart disease (patent ductus arteriosus) with (2 F) corresponding time-frequency diagram where leads V3 - V5 exhibit signals of highest intensity

This preprocessing workflow was meant to ensure high-quality spectral representations of ECG signals and optimize the data for machine learning analyses. Representative examples of ECG waveforms and their corresponding time–frequency spectrograms are illustrated in Fig. 2.

### Signal preprocessing [21, 22]

To ensure high-quality ECG signal processing, the following pre-processing techniques were applied:

#### Signal pre-processing:

1. *Baseline wander removal:*
  Baseline drift, typically caused by patient movement or respiration, was removed using a high-pass filter with a cutoff frequency of 0.5 Hz.
  This eliminated slow-varying components, while preserving diagnostically relevant QRS and ST segments.
2. *Powerline interference reduction:*
  AC noise contamination due to powerline interference (60 Hz in Taiwan) was suppressed by applying a notch filter centered at 60 Hz.
3. *Low-pass filtering:*
  High-frequency noise was attenuated using a low-pass filter with a cutoff frequency of 100 Hz to eliminate high-frequency artifacts while preserving ECG components of clinical relevance.
4. *Segmentation:*
  Each 10-second ECG recording was divided into five non-overlapping 2-second segments to enhance data diversity and improve model training.
  Each segment retained the original 500 Hz sampling rate, forming a 12 × 1000 matrix (12 leads × 1000 time points).
5. *Continuous Wavelet Transform (CWT):*
  The CWT of a signal $x\;(t)$ is defined as follows:
  $$W\left(a,b\right)={\int }_{-\infty }^{\infty }x\left(t\right){\psi }^{*}\left(\frac{t-b}{a}\right)dt$$
  where $W\;(a,\;b)$ represents the wavelet coefficient at scale a and position b; $a\;>\;0$ is the scale parameter controlling the dilation or compression of the wavelet; $b\;\in\;\mathbb{R}$ is the translation parameter determining the shift along the time axis; and $\psi\;(t)$ is the mother wavelet function, and $\psi\ast\;(t)$ denotes its complex conjugate.
  Morlet Wavelet (Mother Wavelet):
  This study selected the Morlet wavelet due to its ability to balance time and frequency localization in the analysis of ECG signals. It is expressed as
  $$\psi\;(t)\;=\;e^{j2\pi f_0t}e^\frac{-t^2}{{2\sigma}^2}$$
  where $f_0$ is the central frequency of the wavelet; $\sigma$ determines the time-domain spread; and *t* represents time.
6. Normalization:
  CWT spectrogram values were normalized to a range of [0, 1] to facilitate stable and efficient model training.
7. *Artifact removal:*
  Segments with excessive noise or loss of lead contact (e.g., flatline or irregular spikes) were manually excluded by an experienced technician prior to feature extraction.

### Model training

MATLAB 2022b (Natick, Massachusetts, USA) was utilized for model training. Transfer learning was implemented using three pre-trained convolutional neural networks (CNNs): ResNet- 18 [23], InceptionResNet-V2 [24], and NasNetMobile [25]. The third-to-last fully connected layer of each model underwent output size modification, and the final classification layer was replaced to accommodate the patient groups in this study.

To ensure a balanced dataset, 80% of the data were allocated to the training set, while the remaining 20% were designated as the test set. To maintain consistency across sets, we calculated the modulo of our data, ensuring that all ECG samples from the same patient were assigned to the same set.

Below, we outline the hyperparameter settings employed in the training of ResNet- 18, InceptionResNet-V2, and ASNetMobile as well as the rationale behind their selection.

- Optimizer: We employed the Stochastic Gradient Descent with Momentum (SGDM) as an optimizer, as it provides a good balance between stability and convergence speed. It is widely used in medical image and signal processing tasks involving deep learning.
- Initial learning rate = 0.001: This is a common setting for transfer learning, allowing fine-tuning of pre-trained models without drastic parameter updates.
- Momentum = 0.9: This value was selected to accelerate convergence by dampening oscillations during gradient updates.
- L2 regularization = 0.1: This value was selected to prevent overfitting, considering the relatively small size of our image dataset.
- Minibatch size = 8: This value is meant to balance computational efficiency and convergence stability under GPU memory constraints.
- Training = 5 epochs: We limited the number of training epochs because the models had been pre-trained on large datasets, and experiments revealed a plateau in performance after a few epochs, suggesting that prolonged training could lead to overfitting.
- Validation: five-fold cross-validation was employed to ensure robust model performance without excessive dependence on any particular data partition.

**Fig. 3:**
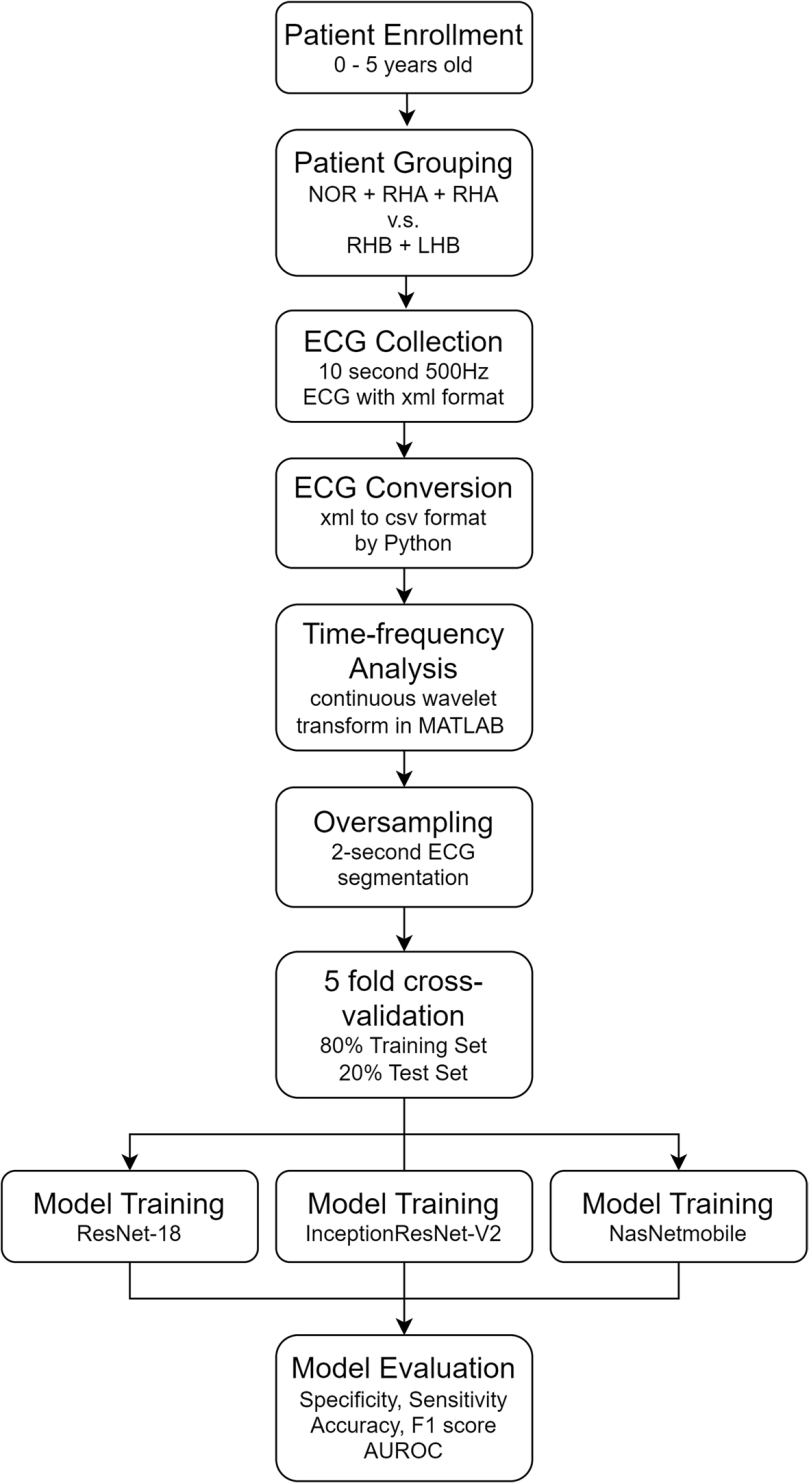
Process of training and classification: 10-second ECGs were first exported from MUSE in XML format for conversion into CSV format within a Python environment. The CSV files were imported into MATLAB to undergo continuous wavelet transform. Oversampling was performed by segmenting the initial ECG segments into fie 2-second intervals. The dataset was then split into a training set (80% of the ECG data) and a test set (the remaining 20%). Model training and 5-fold cross-validation were conducted using three pre-trained architectures: ResNet- 18, InceptionResNet-V2, and NasNetMobile. Performance was evaluated in terms of accuracy, sensitivity, specificity, F1 score, and area under the ROC curve (AUC)

Figure 3 Outlines the preprocessing and training workflow. All computation was performed using a custom-assembled workstation, equipped with an Intel Core i9 - 13900 K CPU, 128GB of RAM, and an Nvidia GeForce RTX 4090 GPU to ensure high-performance.

### Model evaluation

Model performance was assessed by comparing the predicted results with clinical reports generated by a pediatric cardiologist. According to rule based criteria proposed by Society Guideline [26, 27], an ECG was classified as abnormal if the clinical report indicated the presence of atrial or ventricular hypertrophy.

### Statistical analysis

After model training, confusion matrices were generated to assess the performance of our model using the following metrics: accuracy, sensitivity, specificity, F1 score, and area under the receiver operating characteristic curve (AUC). The performance metrics are based on four components from the confusion matrix used in binary classification: true positive (TP), true negative (TN), false positive (FP), and false negative (FN). The formulas used to derive the performance metrics are as follows:

$$\begin{array}{c}Accuracy=\frac{True\; positive\left(TP\right)+True\;negative(TN)}{TP+TN+false\;positive\left(FP\right)+false\;negative(FN)}\\Sensitivity=\frac{TP}{TP+FN}\\Specificity=\frac{TN}{TN+FP}\\F1score=\frac{2TP}{2TP+FP+FN}\end{array}$$

The results are reported as the mean, standard deviation, and 95% confidence interval derived from five-fold cross-validation. All statistical analysis was performed using MATLAB 2022b and Microsoft Excel.

### Ethical approval

All methods in this study were performed in strict accordance with the Declaration of Helsinki. This study was approved by Institutional Review Board (IRB) of Chang Gung Medical Foundation (Reference number: 202102195B0). Due to retrospective design of this study, the IRB committee waived the need for participant consent.

## Results

This study examined 1,035 patients aged 0 to 5, including 234 (22%) with normal heart structure (NOR), 100 patients (10%) with non-significant right heart disease (RHA), 291 patients (28%) with significant right heart disease (RHB), 141 patients (14%) with non-significant left heart disease (LHA), and 269 patients (26%) with significant left heart disease (LHB).

The age distribution was as follows: 0 years old (416 patients; 40.2%), 1 year (249 patients; 24.1%), 2 years (164 patients; 15.8%), 3 years (99 patients; 9.6%), and 4 years (107 patients; 10.3%). Table 3 lists the distribution of case numbers and the mean age in each group.

**Table 3:** Patient distribution and mean age by group

	NOR	RHA	RHB	LHA	LHB
Case Number	234	100	291	141	269
Mean ± STD (95% CI) (m)	20.6 ± 19.0 (18.2–23.0)	16.5 ± 15.3 (13.4 + 19.5)	24.4 ± 18.8 (22.2–26.6)	19.6 ± 13.2 (17.3–21.7)	16.1 ± 15.2 (14.3–18.0)

*STD* standard deviation, *CI* confidence interval

**Fig. 4:**
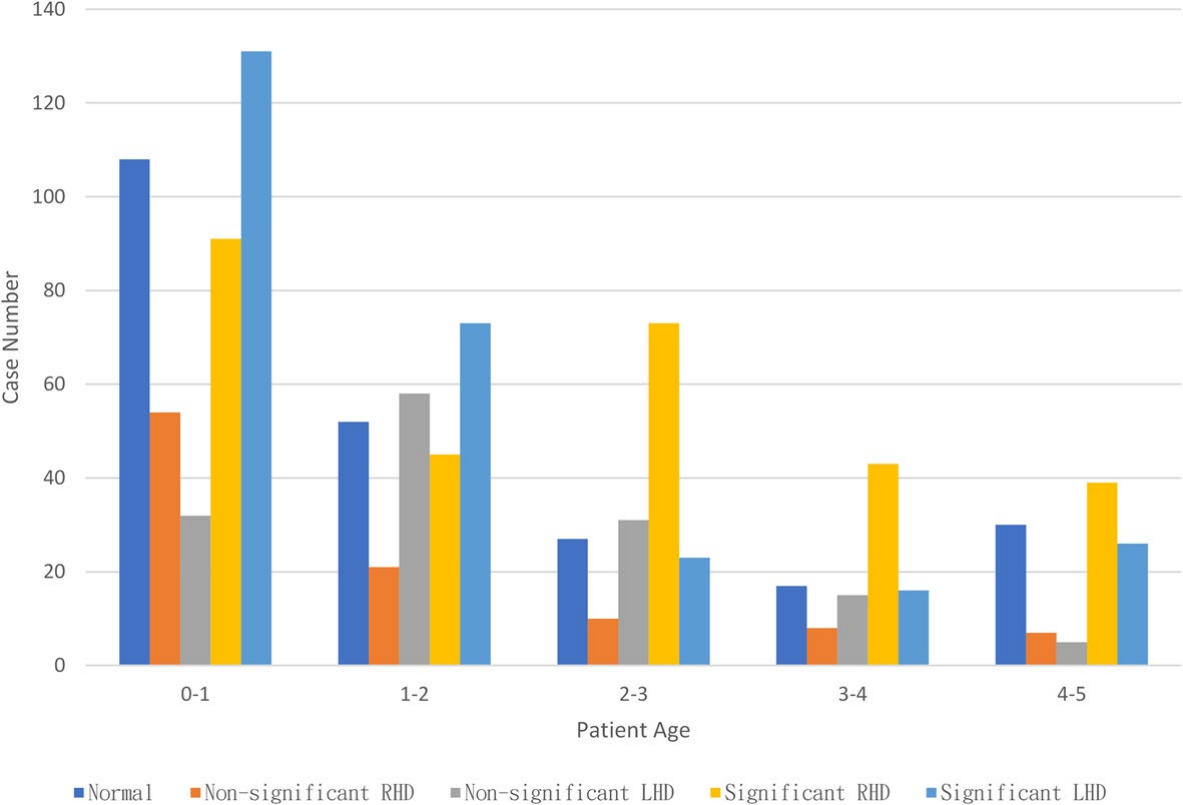
Heart defect and age distributions of patients. The cohort comprised examined 1,035 patients aged 0 to 5, including 234 (22%) with normal heart structure (NOR), 100 patients (10%) with non-significant right heart disease (RHA), 291 patients (28%) with significant right heart disease (RHB), 141 patients (14%) with non-significant left heart disease (LHA), and 269 patients (26%) with significant left heart disease (LHB). The age distribution was as follows: 0 years old (416 patients; 40.2%), 1 year (249 patients; 24.1%), 2 years (164 patients; 15.8%), 3 years (99 patients; 9.6%), and 4 years (107 patients; 10.3%)

The mean age varied across groups, with the highest mean age in the RHB group (24.4 ± 18.8 months, 95% CI: 22.2–26.6 months) and the lowest mean age in the LHB group (16.1 ± 15.2 months, 95% CI: 14.3–18.0 months). The NOR and LHA groups presented similar age distributions, while the mean age in the RHA group was slightly lower than in the NOR group (ANOVA P < 0.05). Figure 4 illustrates the distributions of heart disease types and ages, and Table 4 presents a detailed breakdown of heart defects in the dataset.

**Table 4:** Demographic data

Age	0–1	1–2	2–3	3–4	4–5	Subtotal	Ratio
Normal heart (NOR)	108	52	27	17	30	234	
Ratio	0.462	0.222	0.115	0.073	0.128	1.000	
Non-significant right heart disease (RHA)							
ASD	48	17	6	7	5	83	0.830
PS	6	4	4	1	2	17	0.170
Subtotal	54	21	10	8	7	100	1.000
Ratio	0.540	0.210	0.100	0.080	0.070	1.000	
Non-significant left heart disease (LHA)							
VSD	18	12	4	4	2	40	0.284
PDA	12	44	25	10	2	93	0.660
AS	1	2	1	1	1	6	0.043
CoA	1	0	1	0	0	2	0.014
Subtotal	32	58	31	15	5	141	1.000
Ratio	0.227	0.411	0.220	0.106	0.035	1.000	
Significant right heart disease (RHB)							
ASD	19	20	63	33	29	164	0.564
PS	44	7	4	6	8	69	0.237
TOF	28	18	6	4	2	58	0.199
Subtotal	91	45	73	43	39	291	1.000
Ratio	0.313	0.155	0.251	0.148	0.134	1.000	
Significant left heart disease (LHB)							
VSD	84	38	13	11	20	166	0.617
PDA	37	30	6	3	1	77	0.286
AS	8	5	3	1	4	21	0.078
CoA	2	0	1	1	1	5	0.019
Subtotal	131	73	23	16	26	269	1.000
Ratio	0.487	0.271	0.086	0.059	0.097	1.000	
Age	0–1	1–2	2–3	3–4	4–5	Total	
	416	249	164	99	107	1035	
Ratio	0.402	0.241	0.158	0.096	0.103	1.000	
					Total	1035	

In detecting total right heart disease (NOR vs RHA + RHB), the model derived from InceptionResNet-V2 presented the best overall performance, with accuracy of 0.707 ± 0.027 (95% CI: 0.656—0.758), F1 score of 0.772 ± 0.035 (95% CI: 0.704—0.773), and AUC of 0.758 ± 0.027 (95% CI: 0.725—0.791). In detecting clinically significant right heart disease (NOR + RHA vs RHB), the model derived from ResNet- 18 presented the best performance, with accuracy of 0.789 ± 0.009 (95% CI: 0.778—0.799), F1 score of 0.770 ± 0.014 (95% CI: 0.704—0.773), and AUC of 0.852 ± 0.011 (95% CI: 0.838—0.866).

In detecting total left heart disease (NOR vs LHA + LHB), the model derived from InceptionResNet-V2 presented the best performance, with accuracy of 0.710 ± 0.011 (95% CI: 0.697—0.724), F1 score of 0.737 ± 0.008 (95% CI: 0.727—0.747), and AUC of 0.802 ± 0.019 (95% CI: 0.802 ± 0.019). In detecting significant left heart disease (NOR + LHA vs LHB), the model derived from InceptionResNet-V2 presented the best performance, with accuracy of 0.744 ± 0.033 (95% CI: 0.704—0.785), F1 score of 0.695 ± 0.035 (95% CI: 0.652—0.738), and AUC of 0.816 ± 0.035 (95% CI: 0.773—0.859). The results are detailed in Table 5.

**Table 5:** Impact of significance in prediction of CHD: results of transfer learning using various pre-trained models

NOR vs RHA + RHB			
	ResNet- 18	InceptionResNet-V2	NASNetMobile
Sensitivity	0.719 ± 0.029 (0.683–0.755)	0.804 ± 0.049 (0.742–0.865)	0.750 ± 0.041 (0.699–0.800)
Specificity	0.633 ± 0.044 (0.579–0.688)	0.551 ± 0.051 (0.488–0.615)	0.609 ± 0.078 (0.512–0.706)
Accuracy	0.686 ± 0.033 (0.645–0.727)	0.707 ± 0.041 (0.656–0.758)	0.696 ± 0.012 (0.681–0.711)
F1 score	0.739 ± 0.028 (0.704–0.773)	0.772 ± 0.035 (0.728–0.816)	0.752 ± 0.011 (0.738–0.767)
AUROC	0.738 ± 0.029 (0.702–0.774)	0.758 ± 0.027 (0.725–0.791)	0.757 ± 0.022 (0.730–0.785)
NOR + RHA vs RHB			
	ResNet- 18	InceptionResNet-V2	NASNetMobile
Sensitivity	0.735 ± 0.032 (0.695–0.775)	0.759 ± 0.052 (0.695–0.824)	0.747 ± 0.074 (0.655–0.839)
Specificity	0.839 ± 0.022 (0.812–0.865)	0.803 ± 0.055 (0.734–0.872)	0.824 ± 0.043 (0.770–0.878)
Accuracy	0.789 ± 0.009 (0.778–0.799)	0.782 ± 0.036 (0.737–0.827)	0.787 ± 0.019 (0.764–0.811)
F1 score	0.770 ± 0.014 (0.753–0.787)	0.770 ± 0.040 (0.721–0.819)	0.770 ± 0.034 (0.728–0.811)
AUROC	0.852 ± 0.011 (0.838–0.866)	0.867 ± 0.020 (0.842–0.892)	0.865 ± 0.020 (0.840–0.890)
NOR vs LHA + LHB			
	ResNet- 18	InceptionResNet-V2	NASNetMobile
Sensitivity	0.617 ± 0.037 (0.571–0.663)	0.640 ± 0.018 (0.618–0.661)	0.547 ± 0.037 (0.501–0.592)
Specificity	0.857 ± 0.044 (0.803–0.911)	0.834 ± 0.045 (0.778–0.890)	0.895 ± 0.031 (0.856–0.934)
Accuracy	0.704 ± 0.010 (0.691–0.717)	0.710 ± 0.011 (0.697–0.724)	0.673 ± 0.017 (0.652–0.695)
F1 score	0.726 ± 0.017 (0.704–0.747)	0.737 ± 0.008 (0.727–0.747)	0.680 ± 0.025 (0.649–0.711)
AUROC	0.817 ± 0.021 (0.790–0.843)	0.802 ± 0.019 (0.777–0.826)	0.786 ± 0.025 (0.754–0.817)
NOR + LHA vs LHB			
	ResNet- 18	InceptionResNet-V2	NASNetMobile
Sensitivity	0.626 ± 0.060 (0.551–0.701)	0.696 ± 0.038 (0.649–0.744)	0.481 ± 0.022 (0.454–0.509)
Specificity	0.815 ± 0.014 (0.797–0.832)	0.779 ± 0.043 (0.725–0.832)	0.912 ± 0.013 (0.895–0.929)
Accuracy	0.736 ± 0.033 (0.695–0.777)	0.744 ± 0.033 (0.704–0.785)	0.732 ± 0.008 (0.721–0.742)
F1 score	0.664 ± 0.050 (0.602–0.725)	0.695 ± 0.035 (0.652–0.738)	0.600 ± 0.016 (0.580–0.620)
AUROC	0.804 ± 0.031 (0.765–0.842)	0.816 ± 0.035 (0.773–0.859)	0.814 ± 0.017 (0.793–0.835)

Mean ± SD (95% CI)

*SD* standard deviation, *CI* confidence interval

**Fig. 5:**
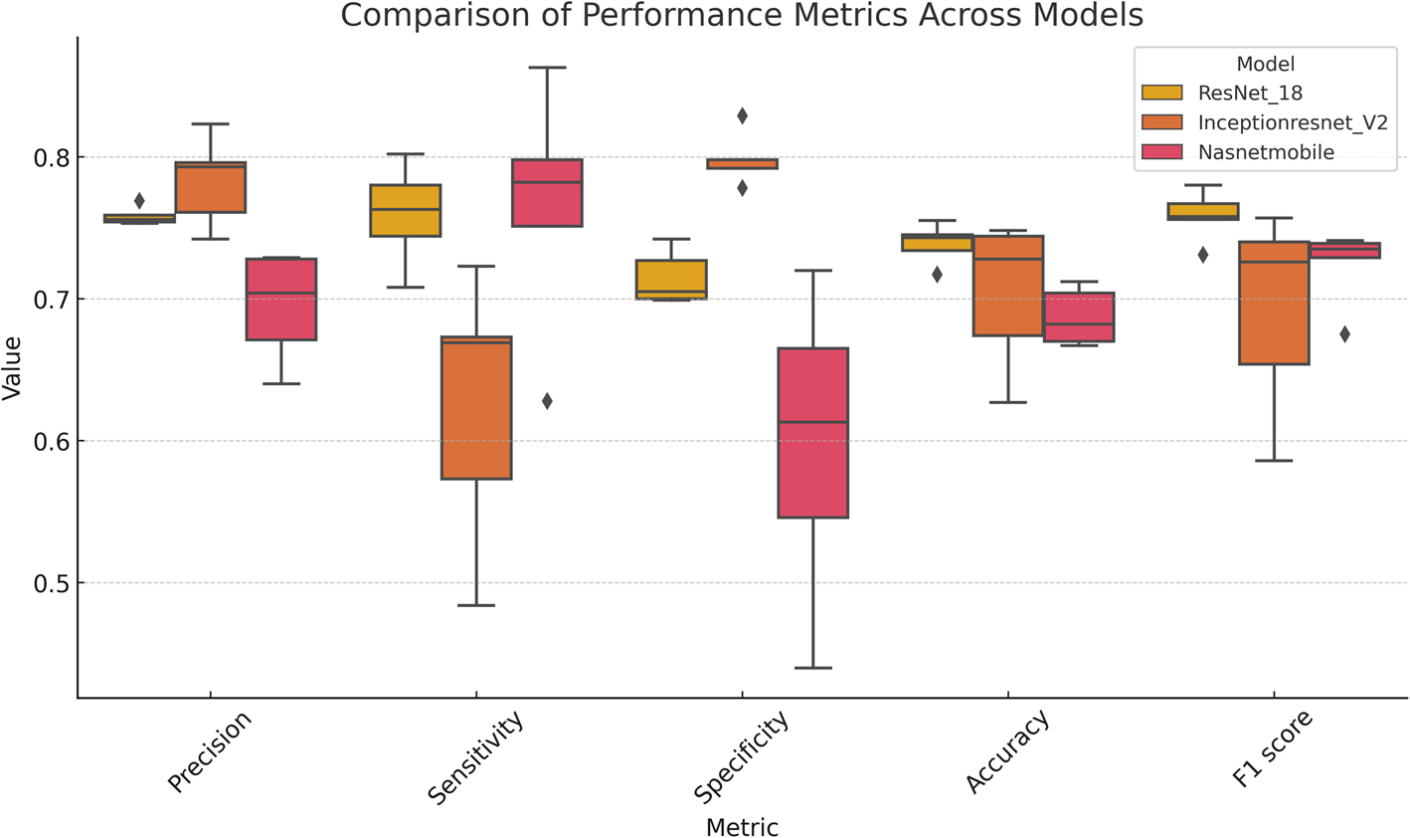
Boxplots illustrating 5-fold cross-validation results in predicting clinically significant CHD. The Nasnet- 18 model demonstrated the best detection performance across all metrics. InceptionResNetV2 also demonstrates strong performance, however it presented variability in sensitivity and F1-scores

To simulate the conditions typically encountered in daily practice, we combined right and left heart diseases into one group. In this analysis, the model derived from ResNet- 18 achieved the best performance, with accuracy of 0.739 ± 0.012 (95% CI: 0.724—0.753), F1 score of 0.758 ± 0.015 (95% CI: 0.740—0.776), and AUC of 0.810 ± 0.013 (0.794—0.825). Figure 5 presents a boxplot for these results of five-fold cross-validation. Comprehensive loss curves are presented in Supplementary Fig. 1.

The average elapsed time for model training was as follows: InceptionResNet V2 (33 min 12 secs), ResNet- 18 (7 min 27 secs, and NasNetMobile (51 min 46 secs). These results are detailed in Table 6.

**Table 6:** Result of clinically-applicable model (NOR + RHA + LHA vs. RHB + LHB)

	Sensitivity	Specificity	Accuracy	F1 score	AUC
ResNet- 18	0.759 ± 0.029 (0.723—0.795)	0.715 ± 0.015 (0.695—0.734)	0.739 ± 0.012 (0.724—0.753)	0.758 ± 0.015 (0.740—0.776)	0.810 ± 0.013 (0.794—0.825)
InceptionResNet-V2	0.624 ± 0.078 (0.528—0.721)	0.799 ± 0.015 (0.780—0.818)	0.704 ± 0.043 (0.651—0.758)	0.693 ± 0.058 (0.620—0.765)	0.791 ± 0.028 (0.757—0.826)
NASNetMobile	0.765 ± 0.071 (0.677—0.852)	0.597 ± 0.089 (0.486—0.707)	0.687 ± 0.017 (0.666—0.707)	0.724 ± 0.023 (0.696—0.752)	0.771 ± 0.020 (0.746—0.796)

Mean ± SD (95% CI)

*SD* standard deviation, *CI* confidence interval, *NOR* normal, *RHA* non-significant right heart disease, *LHA* non-significant left heart disease, *RHB* significant right heart disease, *LHB* significant left heart disease, *AUC* area under curve

The performance metrics in the ECG reports generated by a pediatric cardiologist for the current dataset were as follows: accuracy 0.671, sensitivity 0.716, specificity 0.648, F1 score 0.702. Overall, the proposed AI model proved superior to current best practices in screening for clinically significant congenital heart disease based on ECG vector changes.

## Discussion

This study demonstrated the application of deep learning model derived from ResNet- 18 for the classification of congenital heart disease (CHD) based on ECG data, achieving a good balance between performance and computational efficiency. Notably, our ResNet- 18 model outperformed InceptionResNet-V2 in overall performance due to its efficient architecture and generalizability across CHD subtypes. Our findings demonstrate the potential utility of AI-enhanced ECG interpretation as a screening tool for hemodynamically significant CHD in infants and young children.

### Scope of CHD inclusion and model generalizability

A universal screening tool should ideally detect all forms of CHD, including rare but critical conditions (e.g., single ventricle defects, hypoplastic left heart syndrome, Ebstein’s anomaly). However, our primary focus was on the detection of hemodynamically significant acyanotic CHD in infants and young children—a common but underdiagnosed subgroup—due to the following reasons:

- Clinical prevalence: Left-to-right shunt lesions (e.g., ventricular septal defect, atrial septal defect, patent ductus arteriosus) and obstructive lesions (e.g., coarctation of the aorta, aortic stenosis) constitute the majority of CHD cases in this age group.
- Screening utility: Acyanotic but hemodynamically significant defects often evade detection by pulse oximetry screening, as their initial presentation tends to subtle or asymptomatic. Delayed diagnosis increases the risk of heart failure.
- Common ECG features: The ECG abnormalities observed in both common and rare congenital heart diseases stem primarily from vector changes. In right heart disease, these changes manifest as right axis deviation and right ventricular hypertrophy, whereas in left heart disease, they appear as left axis deviation and left ventricular hypertrophy.

The authors suspect that our AI model could detect even rare, life-threatening congenital heart diseases, provided they exhibit pronounced ECG changes. The generalizability of the model could likely be improved by increasing the number of normal ECGs beyond the 234 included in this study. Furthermore, a prospective study incorporating ECGs from healthy pediatric patients would better reflect real-world screening populations.

### Performance of proposed model in differentiating left and right heart disease

The model derived from ResNet- 18 demonstrated the best overall performance in detecting right heart disease, which is consistent with previous studies that used ECG to detect right ventricular hypertrophy. However, its performance was significantly lower when applied to left heart disease. This suboptimal performance may be attributed to developmental factors, such as the rapid increase in left ventricular (LV) mass early in life, which can obscure ECG markers of left heart disease. Another possible explanation is the difficulty in feature extraction, as detection of left heart disease traditionally relies on echocardiographic parameters, and ECG findings—such as left axis deviation—are often subtle and lack reliability.

### Detection of ECG abnormalities

One previous study on the detection of ventricular hypertrophy in right heart diseases reported accuracy of 0.78, which is comparable to the performance of our ResNet- 18 model (0.79). Another study using ECG rule-based criteria for detecting left ventricular hypertrophy achieved relatively low accuracy (0.65–0.75) [28]. The performance of our AI-based method was also comparable to a prior study that employed similar techniques for detecting atrial septal defect (ASD), achieving an AUC of 0.88 [8].

Rule-based ECG classifications frequently fall within the normal range, making them less reliable for ruling out congenital heart disease (CHD). As a result, it is unreasonable to expect clinicians to diagnose CHD solely based on ECG findings. In this study, left ventricular hypertrophy (LVH) and right ventricular hypertrophy (RVH) were used as benchmarks to ensure alignment with standard clinical practice.

### Model design, hyperparameters, and oversampling strategy

In this study, ResNet- 18 outperformed InceptionResNet-V2 and NasNetMobile, due to its efficient residual connections and relatively low computational complexity. Many studies on the application of AI techniques to the interpretation of ECG data employ pretrained models utilizing residual networks [12, 29]. In the current study, ResNet- 18 achieved accuracy on par with deeper models, such as ResNet- 50 and ResNet- 101, while requiring significantly less training time, making it a better alternative for real-world deployment.

The pre-trained Inception model, developed by Google Research, has been previously applied to AI research on CHD [7, 30]. In this study, we selected its latest iteration, which emphasizes residual connections for enhanced performance. We also utilized the NasNetMobile pre-trained model, which differs from manually engineered architectures by employing neural architecture search (NAS) to optimize network design. NasNetMobile is specifically tailored for mobile devices, ensuring efficient performance in low-power environments, such as medical offices where computational resources tend to be limited.

CHD datasets often suffer from class imbalances due to the low prevalence of certain conditions. To address this issue, we implemented an oversampling strategy, in which each 10-s ECG recording was segmented into five 2-s segments. This segmentation approach allowed us to generate a more balanced dataset, reducing the risk of the model being biased toward the majority class.

To mitigate concerns regarding temporal dependencies, we applied Continuous Wavelet Transform (CWT), which captures information in both the time and frequency domains, minimizing potential distortion.

Future research will explore additional data augmentation techniques and alternative approaches to segmentation.

### Clinical significance and future implications

It is important to consider that the expertise in pediatric cardiology tends to be concentrated in large medical centers. Thus, the proposed AI model could potentially expand CHD screening into resource-limited areas. AI-augmented ECG analysis could serve as a supplementary tool for early CHD detection, similar to the way pulse oximetry testing is used to screen newborn. Possible implementations include the following:

- Telemedicine integration: AI models could facilitate remote ECG analysis, reducing the need for in-person evaluations by specialists.
- Primary care utility: Clinicians without specialized training in cardiology could use AI-assisted ECG screening to identify at-risk infants who require echocardiography.
- Integration with other modalities: Previous studies have demonstrated that a combination of AI-based ECG analysis with human intervention can enhance detection performance [31]. Moreover, chest X-ray (CXR) imaging has been used to evaluate the hemodynamic significance of CHD [32]. Integrating multiple modalities could lead to the development of a versatile screening models.

This study was subject to several limitations, which should be considered in the interpretation of our findings. First, the retrospective nature of this study and its reliance on single-center data highlight the need for future multi-center studies to verify the generalizability of the model. Moreover, the lack of an external test set means that further external validation will be required to assess its real-world applicability.

Another limitation is the inclusion of only a few rare CHD cases, which restricted the ability of the model to generalize across a broad spectrum of congenital heart conditions. Expanding the dataset to include more diverse CHD subtypes would improve model robustness. It is also important to consider the potential ECG acquisition bias, as differences in operator techniques and device settings may impact model performance. Future studies should evaluate the model using ECG records acquired by multiple operators under various operating conditions to ensure its reliability across different clinical settings.

Lastly, the influence of age-related changes on ECG interpretation suggests a need for further age-stratified analysis. Developing age-specific models could enhance diagnostic accuracy, particularly for younger patients with ECG markers that vary significantly with cardiac maturation.

## Conclusion

This study marks a significant advancement in AI-assisted CHD screening for young children. Our ResNet- 18-based model demonstrated stable performance in the detection of hemodynamically significant CHD, effectively balancing accuracy and computational efficiency. The proposed AI-driven model outperformed conventional ECG-based screening methods that rely on rule-based criteria. When used as a complement to pulse oximetry screening in newborns, this approach could facilitate early detection of conditions requiring intervention, thereby reducing the risk of complications in children aged 0 to 5, a period of rapid cardiac development.

## Supplementary Information

Supplementary Material 1: Supplemental Figure 1. Representative loss curve for the prediction model utilizing the ResNet- 18 pre-trained model

## Data Availability

Data collected in the current study are available from the corresponding author upon reasonable request.

## Abbreviations

AS: Aortic valve stenosis
ASD: Atrial septal defect
CHD: Congenital heart disease
CoA: Coarctation of aorta.
ECG: Electrocardiogram
LHA: Non-significant left heart disease
LHB: Significant left heart disease
NOR: Normal heart structure
PDA: Patent ductus arteriosus
PS: Pulmonary valve stenosis
RHA: Non-significant right heart disease
RHB: Significant right heart disease
SGDM: Stochastic gradient descent with momentum
TOF: Tetralogy of Fallot
VSD: Ventricular septal defect
