# Supplementary figures and images for: Prediction of significant congenital heart disease in infants and children using continuous wavelet transform and deep convolutional neural network with 12-lead electrocardiogram
Source: BMC Pediatr. 2025 Apr 24;25:324. doi: 10.1186/s12887-025-05628-2 (PMC12020324; doi:10.1186/s12887-025-05628-2)

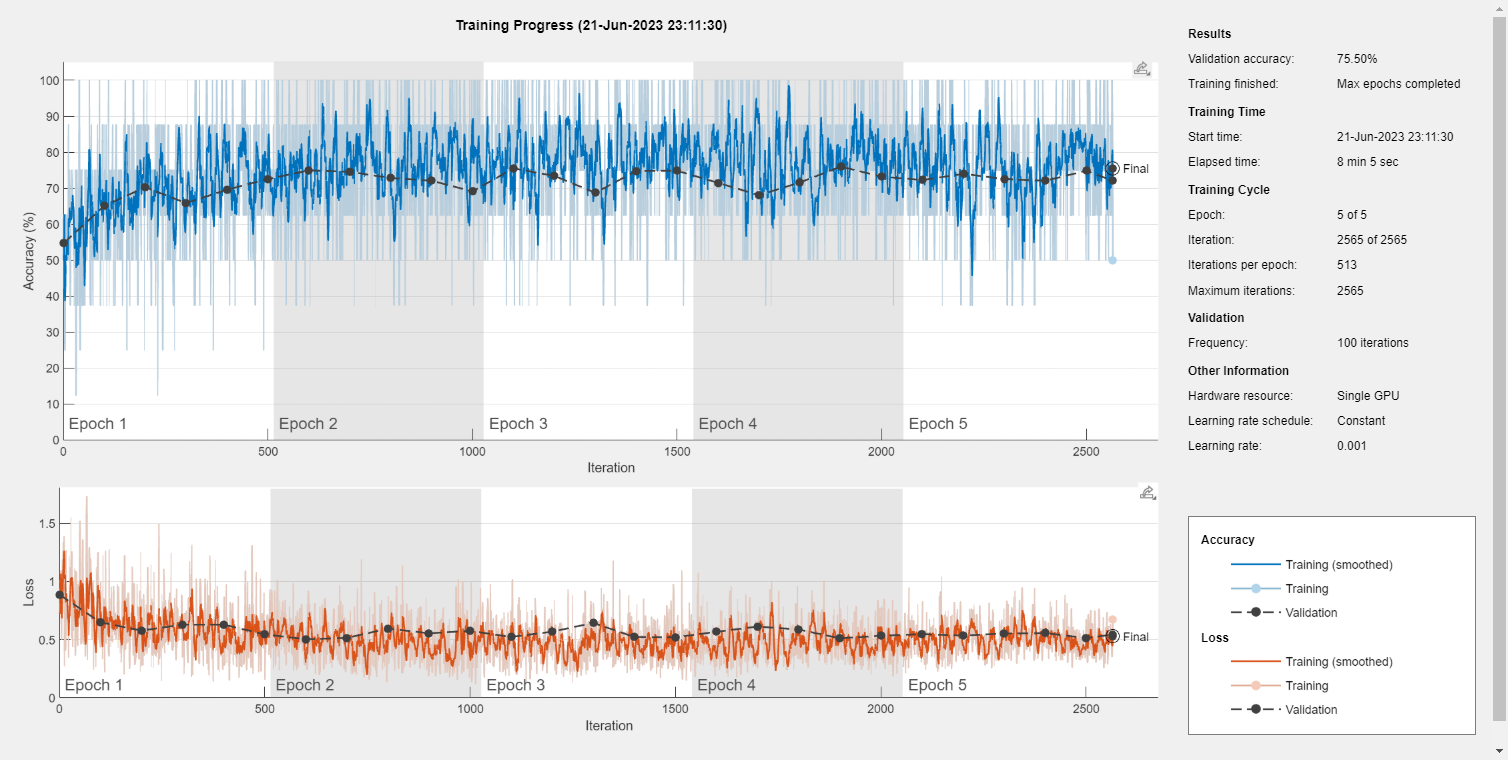

Supplement: Supplementary file 1 — Supplementary Material 1: Supplemental Figure 1. Representative loss curve for the prediction model utilizing the ResNet- 18 pre-trained model [file 12887_2025_5628_MOESM1_ESM.png]
